# A Vaccine Therapy for Canine Visceral Leishmaniasis Promoted Significant Improvement of Clinical and Immune Status with Reduction in Parasite Burden

**DOI:** 10.3389/fimmu.2017.00217

**Published:** 2017-03-07

**Authors:** Bruno Mendes Roatt, Rodrigo Dian de Oliveira Aguiar-Soares, Levi Eduardo Soares Reis, Jamille Mirelle de Oliveira Cardoso, Fernando Augusto Siqueira Mathias, Rory Cristiane Fortes de Brito, Sydnei Magno da Silva, Nelder De Figueiredo Gontijo, Sidney de Almeida Ferreira, Jesus G. Valenzuela, Rodrigo Corrêa-Oliveira, Rodolfo Cordeiro Giunchetti, Alexandre Barbosa Reis

**Affiliations:** ^1^Laboratório de Imunopatologia, Núcleo de Pesquisas em Ciências Biológicas, Universidade Federal de Ouro Preto, Ouro Preto, Minas Gerais, Brazil; ^2^Departamento de Patologia Clínica, COLTEC, Universidade Federal de Minas Gerais, Belo Horizonte, Minas Gerais, Brazil; ^3^Instituto Nacional de Ciência e Tecnologia em Doenças Tropicais (INCT-DT), Salvador, Brazil; ^4^Laboratório de Bioensaios em Leishmania, Instituto de Ciências Biomédicas, Universidade Federal de Uberlândia, Uberlândia, Minas Gerais, Brazil; ^5^Laboratório de Fisiologia de Insetos Hematófagos, Departamento de Parasitologia, Universidade Federal de Minas Gerais, Belo Horizonte, Minas Gerais, Brazil; ^6^Vector Molecular Biology Section, Laboratory of Malaria and Vector Research, National Institute of Allergy and Infectious Diseases, National Institutes of Health, Rockville, MD, USA; ^7^Laboratório de Biologia das Interações Celulares, Departamento de Morfologia, Universidade Federal de Minas Gerais, Belo Horizonte, Minas Gerais, Brazil

**Keywords:** heterologous vaccine therapy, immunotherapy, canine visceral leishmaniasis, *L. infantum*, immune response

## Abstract

Herein, we evaluated the treatment strategy employing a therapeutic heterologous vaccine composed of antigens of *Leishmania braziliensis* associated with MPL adjuvant (LBMPL vaccine) for visceral leishmaniasis (VL) in symptomatic dogs naturally infected by *Leishmania infantum*. Sixteen dogs received immunotherapy with MPL adjuvant (*n* = 6) or with a vaccine composed of antigens of *L. braziliensis* associated with MPL (LBMPL vaccine therapy, *n* = 10). Dogs were submitted to an immunotherapeutic scheme consisting of 3 series composed of 10 subcutaneous doses with 10-day interval between each series. The animals were evaluated before (T0) and 90 days after treatment (T90) for their biochemical/hematological, immunological, clinical, and parasitological variables. Our major results showed that the vaccine therapy with LBMPL was able to restore and normalize main biochemical (urea, AST, ALP, and bilirubin) and hematological (erythrocytes, hemoglobin, hematocrit, and platelets) parameters. In addition, in an *ex vivo* analysis using flow cytometry, dogs treated with LBMPL vaccine showed increased CD3^+^ T lymphocytes and their subpopulations (TCD4^+^ and TCD8^+^), reduction of CD21^+^ B lymphocytes, increased NK cells (CD5^−^CD16^+^) and CD14^+^ monocytes. Under *in vitro* conditions, the animals developed a strong antigen-specific lymphoproliferation mainly by TCD4^+^ and TCD8^+^ cells; increasing in both TCD4^+^IFN-γ^+^ and TCD8^+^IFN-γ^+^ as well as reduction of TCD4^+^IL-4^+^ and TCD8^+^IL-4^+^ lymphocytes with an increased production of TNF-α and reduced levels of IL-10. Concerning the clinical signs of canine visceral leishmaniasis, the animals showed an important reduction in the number and intensity of the disease signs; increase body weight as well as reduction of splenomegaly. In addition, the LBMPL immunotherapy also promoted a reduction in parasite burden assessed by real-time PCR. In the bone marrow, we observed seven times less parasites in LBMPL animals compared with MPL group. The skin tissue showed a reduction in parasite burden in LBMPL dogs 127.5 times higher than MPL. As expected, with skin parasite reduction promoted by immunotherapy, we observed a blocking transmission to sand flies in LBMPL dogs with only three positive dogs after xenodiagnosis. The results obtained in this study highlighted the strong potential for the use of this heterologous vaccine therapy as an important strategy for VL treatment.

## Introduction

Visceral leishmaniasis (VL) is considered one of the most neglected of all the neglected diseases with endemic nature expanding in many peri-urban and urban areas of Brazil, Latin America, Europe, and other areas of the world (1, 2). VL is considered the most fatal form of the leishmaniasis if left untreated. In an attempt to control the disease, many strategies are used to interrupt parasite transmission, including elimination of seropositive dogs that act as reservoirs, use of insecticides for vector control and systematic treatment of human cases (3, 4). Several studies have shown the importance of the development of vaccines to control the disease (5–8); however, to date there are no vaccines that can be used in control programs against human or canine disease (9). Moreover, available treatment options are still far from a desirable efficacy due the fact that they are either toxic (antimonials and amphotericin B deoxycholate) or expensive (liposomal amphotericin B) (10). The situation is even more complicated by the imminent emergence of resistant parasites to presently available anti-leishmanial drugs, mainly with monotherapy (miltefosine and paromomycin) (11). Thus, there is an urgent need to improve current drugs and promote investment in the discovery of new treatment schemes such as vaccines or immunomodulators (immunotherapy) to promptly induce an effective and protective immune response against the parasite (10).

Immunotherapy was first used against tegumentar leishmaniasis, mainly in cutaneous and mucosal patients in Venezuela by Convit and colleagues (12, 13). In Brazil, Mayrink et al. (14) obtained 98% of clinical cure in patients with cutaneous leishmaniasis using as treatment a vaccine composed of *Leishmania amazonensis* antigens plus Bacillus Calmette–Guerin (BCG) as adjuvant. Few studies have demonstrated the effectiveness of immunotherapy in visceral disease. Badaro and colleagues (15) showed the efficiency of IFN-γ in the treatment of some refractory patients to conventional chemotherapy. The same authors evaluated the combination of IFN-γ plus antimony (immunochemotherapy) demonstrating that this combined therapy was effective in a preliminary trial in patients with refractory visceral and mucosal leishmaniasis to conventional antimonial chemotherapy (15).

More recently, a large number of studies have developed new protocols focused on immunochemotherapy to treat VL. In this context, the murine models have been employed using different strategies such as synthetic bacterial lipopeptide (Pam3cys), monoclonal antibodies (mAbs) against cytokine receptors or cytokines, dendritic cell-based treatment, and vaccines. All of these strategies are combined with chemotherapy using low dose or short course of an effective conventional drug or new candidates (16–20). Dogs are considered one of the most important models for VL studies including vaccines and treatment tests and more recently has become even more relevant for the evaluation of immunotherapy and immunochemotherapy protocols. In this sense, vaccines associated with conventional chemotherapy have been employed demonstrating important results (21–23). Recently, Santiago and colleagues (24) evaluated the immunotherapeutic potential of P-MAPA, an immunomodulator in symptomatic dogs with VL. After this treatment, dogs showed significant improvement in clinical signs, decrease in IL-10, and increase in IL-2 and IFN-γ production, with reduction in cutaneous parasitism, demonstrating the effectiveness of immunotherapy in symptomatic VL disease (24).

In summary, many studies indicate that immunotherapy is a promising strategy for VL treatment mainly by the reestablishment of immunity. Thus, our study aimed to evaluate effect of using a heterologous therapeutic vaccine—LBMPL—composed of *Leishmania braziliensis* antigens plus monophosphoryl lipid A, a detoxified form of the endotoxin lipopolysaccharide, and potent toll-like receptor 4 activator (25), as an immunotherapy strategy for treatment of VL in symptomatic dogs naturally infected by *Leishmania infantum* as experimental model.

## Materials and Methods

### Ethics Statement

The study protocol was approved by the Ethical Committee for the Use of Experimental Animals of the Universidade Federal de Ouro Preto, Ouro Preto, MG, Brazil, under the protocol number 2010/57. All experiments were performed according to the guidelines of the National Institutes of Health, USA. We made all efforts to minimize animal suffering.

### Study Animals, Inclusion Criteria, and Immunotherapy Protocol

In this study, 16 mixed breed adult dogs of both sexes, naturally infected with *L. infantum* and presenting at least three clinical signs related to the canine visceral leishmaniasis (CVL) (weight loss, dermatitis, and lymphadenopathy), were used. They were kindly provided by the owners after signing the informed consent at the time of the animal retrieval by the Center for Zoonosis Control (Centro de Controle de Zoonoses) of Governador Valadares, Minas Gerais, Brazil, an important endemic area for VL. Dogs were maintained in individual enclosed kennels, with access to water and balanced canine feed *ad libitum*, belonging to the Kennel for Drug Trial and Vaccines for Leishmaniasis of the Universidade Federal de Ouro Preto, Minas Gerais, Brazil. They had direct contact with pen mates and received daily exercise in the facility. Moreover, there was environmental enrichment in the form of several toys in the interior of each cage. Furthermore, the animals were subjected to a standard quarantine protocol with broad spectrum anthelmintic and vaccinated against rabies (Tecpar, Curitiba, PR, Brazil) before the initiation of the immunotherapy protocol. The clinical criterion to select VL symptomatic dogs was according to Reis and colleagues (26). Moreover, we defined as inclusion criteria for this study positive serological tests for VL (ELISA and DPP^®^) as the Brazilian Ministry of Health recommends. Besides that, we associate the positive serological results with parasitological tests using bone marrow aspirates to demonstrate parasites in NNN/LIT culture or through real-time PCR. Only animals showing symptoms and positive serological and parasitological tests were included in the study.

Experimental dogs were divided into two groups: a group that received immunotherapy only with monophosphoryl lipid A adjuvant, considered the control group (MPL, *n* = 6) and a group that received immunotherapy using the vaccine composed by *L. braziliensis* promastigote protein associated with MPL as adjuvant (LBMPL, *n* = 10). The antigen component of the LBMPL vaccine was previously evaluated by our research group demonstrating significant results as a prophylactic vaccine in hamster and dog models of VL (5, 6, 27–31). For our study, we proposed to use a protocol used by Mayrink and colleagues that treat leishmaniasis in Brazil using vaccine therapy (14, 32). The animals were subjected to an immunotherapeutic scheme consisting of 3 treatment series, each series composed of 10 doses with increasing concentrations of the vaccine antigen [60–300 μg antigen protein + 5–25 μg of MPL (days 1–5) and 300 μg of antigen protein + 25 μg of MPL (days 6–10) in 1 mL of sterile 0.9% saline in first series; 300 μg antigen protein + 25 μg of MPL in 1 mL of sterile 0.9% saline in second and third series by subcutaneous route and a 10-day interval between each series]. For the MPL group, the adjuvant was used alone following the same scheme described for LBMPL group. The dogs were evaluated before (T0) and 90 days (T90) after immunotherapy (150 days of the total experiment).

### Sample Collection

Peripheral blood, bone marrow, and skin samples were collected from both groups of dogs before the onset of the treatment (day 0 = T0) and 90 days after completion the immunotherapy protocol (day 150 = T90). The skin biopsies were performed with aid of the tranquilizer acepromazine (1.1 mg/kg) and local blockage with 1% xylocaine using a 5 mm “punch.” The skin samples were obtained from the middle portion of the ear and stored at −80°C until processing. Blood samples were collected according to described protocol (5) and stored at room temperature to perform the biochemical/hematological and immunophenotyping tests. Bone marrow samples were collected according to previous protocol (33) and stored at −80°C until processing.

### Biochemical/Hematological Analysis and *Ex Vivo* Immunophenotyping

The absolute count of lymphocytes in each sample was obtained using a BC-2800 VET auto hematology analyzer (Mindray, China). Erythrocytes and leukocytes were quantified using the same equipment, and the differential leukocyte counts were obtained by blood smear analysis after prior staining by routine methods. For normal range values of white blood cells (WBC) and red blood cells (RBC), 45 healthy dogs were used to create a normal range that was used in this study. The biochemical evaluations consisted of the liver function (dosage of the enzymes: alanine transaminase—ALT/GPT, aspartate aminotransferase—AST/GOT, gamma-glutamyl transferase—γ-GT, alkaline phosphatase—ALP, and bilirubin); renal function (urea and creatinine) and proteinogram (total protein, albumin, globulin, and albumin/globulin ratio) tests. For the analysis, an Automatic Biochemical System (CELM SBA-200, Barueri, SP, Brazil) and commercials Labtest Kits (Labtest Diagnostica SA, Lagoa Santa, MG, Brazil) were used following the instructions described by the manufacturer.

The immunophenotyping of leukocytes (*ex vivo*) was performed according to de Almeida Leal et al. (34) with minor modifications. Canine mAbs anti-CD3 PE (clone CA17.2A12), anti-CD4 FITC (clone YKIX302.9), anti-CD8 AF647 (clone YCATE55.9), anti-CD21 PE (clone CA2.1D6), anti-CD14 RPE-Cy5 (TÜK4), anti-CD5 PE (clone YKIX322.3), and anti-human-CD16 RPE-Cy5 (3G8) all purchased from Serotec, USA were used in an indirect immunofluorescence procedure in which pooled normal rat serum (diluted 1:6,000) was employed as the isotopic control. The results were expressed in absolute counts (cell number per cubic meter) through the product of the percentage of positive cells (CD3^+^, CD4^+^, CD8^+^, CD5^−^CD16^+^, and CD21^+^) within gated lymphocytes by absolute lymphocyte counts. The absolute counts for monocytes were obtained through the products of CD14^+^ cells within ungated leukocytes by the selection of the region of interest, based on morphometric and immunophenotypic graphics of distribution CD14/FL3 versus side scatter.

### *In Vitro* Proliferation and Intracellular Cytokines Assays

The soluble *L. infantum* [MHOM/BR/1070/BH46 antigen soluble *Leishmania infantum* antigen (SLiAg)] was prepared as described by Reis and colleagues (35) from promastigotes harvested from stationary phase in liver infusion tryptose cultures. The concentration of protein in the SLiAg solution was adjusted to 1 mg/mL and stored at −80°C until required for assays.

The *in vitro* proliferation assay was performed as described by Roatt and colleagues (5) with some modifications. Briefly, peripheral blood mononuclear cells (PBMCs) were isolated from 20 mL of heparinized blood that had been layered onto 10 mL of Ficoll–Hypaque density gradient (Histopaque^®^ 1.077; Sigma Chemical Co.) and centrifuged at 450 × *g* for 40 min at room temperature. The separated PBMCs were resuspended in RPMI 1640 medium, homogenized, washed twice with RPMI 1640, centrifuged and finally resuspended in RPMI 1640 at 10^7^ cells/mL for labeling using carboxy fluorescein diacetate succinimidyl ester (CFSE, Molecular Probes, USA). Cells were resuspended in pre-warmed PBS plus 0.1% BSA at a final concentration of 1 × 10^7^ cells/mL, and 2 μL of 5 mM stock Cell Trace CFSE solution (Molecular Probes, USA) were added per 1 mL of the cells (final working concentration: 10 mM). After that, cells were incubated at 37°C for 10 min. Staining was quenched by adding 5 mL of ice-cold RPMI 1640 plus 10% FBS for 5 min on ice. The cells were washed three times at 300 × *g*, 7 min. For the mitogenic stimulus assays, 25-μL aliquots of PBMCs (2.5 × 10^5^ cells/well) were added to triplicate wells with 25 μL of phytohemagglutinin (2.5 μg/mL; Sigma-Aldrich Chemie GmbH, Taufkirchen, Germany) and incubated in a humidified 5% CO_2_ atmosphere at 37°C for 3 (mitogenic-stimulated cultures) or 5 days (antigen-stimulated cultures). In order to investigate the immunophenotypic features, PBMCs were cultured in 48-well flat-bottomed tissue culture plates (Costar, Cambridge, MA, USA), with each well containing 650 μL of supplemented RPMI medium. Aliquots (50 μL) of PBMCs (5.0 × 10^5^ cells/well) were added to triplicate wells with 100 μL of SLiAg (25 μg/mL) or 100 μL of RPMI 1640 as control. Incubation was carried out in a humidified 5% CO_2_ atmosphere at 37°C for 5 days. Afterward the cells were removed, washed twice with PBS, and stained at room temperature for 30 min in the dark with anti-CD4 PE (clone YKIX302.9), anti-CD8 AF647 (clone YCATE55.9), all purchased from Serotec, USA. After that, the cells were washed two times at 300 × *g* for 10 min, resuspended, and fixed in FACS fixing solution (10 g/L paraformaldehyde, 10.2 g/L sodium cacodylate, and 6.63 g/L sodium chloride, pH 7.2) before being run on the flow cytometer (FACScalibur—Becton Dickinson, San Jose, CA, USA).

Immunostaining for cell surface markers and intracellular cytokine assay were performed as previously published (34). Briefly, two polypropylene tubes were prepared for each animal; one as a control tube (1 mL RPMI plus 1 mL of whole blood in heparin), and the second one was the antigen-stimulated tube, using SLiAg at final concentration of 25 μg/mL. The tubes were incubated for 12 h and kept at 37°C in an incubator with 5% CO_2_. Brefeldin A (BFA) (Sigma, St. Louis, MO, USA) was added to each tube at a final concentration of 10 μg/mL, and cultures were then incubated to an additional 4 h in an incubator with 5% CO_2_ at 37°C. A tube containing PMA at a final concentration of 25 ng/mL was used as a positive control at final 4 h of incubation as BFA. First staining was performed for monoclonal anti-surface molecules (CD4^+^ and CD8^+^). After resuspension, we proceeded to stain for intracytoplasmic cytokines anti-IFN-γ (clone CC302) and anti-IL-4 (clone CC303) (Serotec Ltd., Oxford, England) in U-bottom 96-well plates. The microtubes were kept at 4°C until the acquisition of counts on the flow cytometer (FACScalibur—Becton Dickinson, San Jose, CA, USA), which evaluated at least 30,000 events per tube. Final data of proliferation and intracytoplasmic cytokine production assays were expressed as indexes, calculated by dividing the percentage of positive cells observed in the SLiAg-stimulated culture by the one observed in paired control unstimulated culture (SLiAg/CC ratio).

### TNF-α and IL-10 Quantification

Cytokine quantification in culture supernatants from PBMCs was performed by capture ELISA using mouse monoclonal anti-canine TNF-α and IL-10 antibodies (mAb) and biotinylated polyclonal goat anti-canine TNF-α and IL-10 (R&D Systems, USA). Wells of 96-well plates (Maxisorb, Nalgene Nunc International, Rochester, NY, USA) were coated with 1.0 μg/mL mAb for cytokine capture. The detection antibodies were used at 1.5 μg/mL. Recombinant canine TNF-α and IL-10 (R&D Systems, USA) were used to generate standard curves. The test reactivity was detected with 3,3′,5,5′-tetramethylbenzidine (Sigma-Aldrich, USA) in accordance with the manufacturer’s instructions. The assays were read on an automatic EL 800G ELISA microplate reader (Bio Tek Instruments, Winooski, VT, USA) with a 450 nm filter.

### Clinical Parameters

We assessed the dogs for clinical signs of VL at each examination comparing before treatment (T0) and after 90 days of treatment (T90). We analyzed systemic signs (lymphadenopathy, apathy, arthritis, and diarrhea) cutaneo-mucosal signs [alopecia, hyperkeratosis, seborrhea, pyodermatitis, ulcers, vasculitis, depigmentation, onychogryphosis, skin erythema, hair loss, and nodule(s)], and ocular signs (conjunctivitis and keratitis). Moreover, the body weight of dogs was evaluated before and after the immunotherapy.

For the clinical score construction, the parameters were classified according to severity on a scale from 0 to 3, as follows: 0, absent; 1, mild; 2, moderate, and 3, severe. The sum of the clinical score was calculated by adding the points given to each of the 17 listed clinical parameters at each examination time. After that, we evaluated the reduction observed after immunotherapy and transformed these values into percentage. The reduction in the clinical score means that there was a decrease in the number and intensity of the clinical signs.

In order to evaluate the splenomegaly, the dogs were submitted to general anesthesia using the combination of 2 mg/kg of xylazine chloridrate (Calmium; União Química Farmacêutica S/A, Brazil) and 11 mg/kg of ketamine chloridrate (Agener; União Química Farmacêutica S/A, Brazil) by intramuscular injection. After that, the spleen was studied with portable ultrasound equipment (SonoSite SonoHeart Elite Superior 180 Plus; SonoSite Inc., USA), and the thickness of the organ was measured. The alterations observed in the spleen were classified from normal spleen to presenting mild, moderate, or severe splenomegaly.

### Xenodiagnosis

Xenodiagnosis was performed as previously described (36) with some modifications, in order to verify the ability of treated dogs to infect the sand flies. Briefly, just before treatment—T0 and 90 days after concluding the immunotherapy (T90), animals were submitted to general anesthesia, and the internal surface of the right ear was shaved. Forty to fifty 4-day-old females of *Lutzomyia longipalpis* were placed in a FleboContainer (37) and allowed to feed on the right ear for 30 min in a dark room. After the blood meal, the sand flies were fed daily with a 50% fructose solution and kept at 25°C for 5 days. At the end of 5 days, the sand flies were frozen, and females were grouped into pools of 10 specimens and stored at −20°C until DNA extraction.

### Quantification of Parasites in Bone Marrow, Skin, and Sand Flies

Parasite load was evaluated using quantitative real-time PCR (qPCR) in bone marrow, skin, and sand flies before (T0) and 90 days (T90) after treatment. Total DNA extraction of the samples was carried out using Wizard SV Genomic DNA Purification System Kit (Promega Corporation, USA) according to the manufacturer’s instructions. The pools of 10 females of sand flies were submitted to DNA extraction as previously described (38). In order to quantify parasite burdens, primers (forward, 5′ TGT CGC TTG CAG ACC AGA TG 3′ and reverse, 5′ GCA TCG CAG GTG TGA GCA C 3′) that amplified a 90-bp fragment of a single-copy gene of DNA polymerase of *L. infantum* (GenBank accession number AF009147) was used in a TaqMan system (39). Standard curves were prepared for each run using known quantities of pGEM^®^-T plasmids (Promega, USA) containing inserts of interest. qPCR reactions were carried out with a final volume of 25 μL of mixture containing 1× TaqMan Universal Master Mix (Applied Biosystems), 20 pmol of the specific primers, 10 pmol of the labeled probe, and 20 ng of DNA. The thermal cycling conditions included an initial incubation for 2 min at 50°C, followed by a 10-min denaturation at 95°C, and 40 cycles at 95°C for 15 s and 60°C for 1 min each. The standard and samples were analyzed in duplicate for each run. To verify the integrity of the bone marrow and skin samples, the same procedure was carried out for GAPDH gene (AB038240) that amplified a 115-bp fragment. The *L. longipalpis* periodicity gene that amplified an 80-bp fragment (GenBank accession no: AF446142) (40) was used with the same procedure to verify the integrity of sand flies’ samples. Reactions were processed and analyzed in an ABI Prism 7500-Sequence Detection System (Applied Biosystems, USA). The results were expressed as the number of amastigotes per milliliter of bone marrow, per microgram of skin or number of promastigotes per dog (total promastigotes in pools of sand flies/dog).

### Statistical Analysis

Statistical analyses were performed using Prism 5.0 software package (Prism Software, Irvine, CA, USA). Normality of the data was demonstrated using a Kolmogorov–Smirnov test for immunophenotypic profiles (*ex vivo*), cytokine production, intracytoplasmatic cytokine, and clinical evaluation. Data were analyzed for statistical differences using paired *t*-test. The results of proliferation response and parasite burden were considered, according to the Kolmogorov–Smirnov test, not normally distributed, and the Wilcoxon test and Mann–Whitney test were used. In all cases, the two-tailed *P* value was used, and the differences were considered significant when *P* values were <0.05.

## Results

### Immunotherapy with LBMPL Vaccine Shows Normalization of the Biochemical and Hemogram in Infected Dogs and an Increase in T Cells, NK Cells, and Monocytes

Based on the hemogram evaluation, WBC did not show important modifications in both groups of dogs, MPL and LBMPL before (T0) and 90 days after immunotherapy (T90) (Table 1). On the other hand, only the LBMPL-treated dogs demonstrated an entire normalization in RBC parameters with restoration of normal values of erythrocytes, hemoglobin, hematocrit, and platelets (Table 2). The biochemical evaluation showed normalization in urea and creatinine (renal function) and GOT, ALP, and bilirubin (liver function) in LBMPL dogs after immunotherapy (Figure S1 in Supplementary Material).

**Table 1:** **WBC of dogs naturally infected by *L. infantum* before and after immunotherapy**.

	WBC (/mm^3^)	Normal range	Timeline after immunotherapy	
			T0	T90
MPL	Leukocytes	7,408–14,440	9,680 ± 2,275	10,800 ± 2,636
	Neutrophils	3,839–9,379	5,353 ± 906	7,474 ± 2,886
	Eosinophils	150–709.4	269 ± 129	670 ± 251^a^
	Lymphocytes	2,299–5,119	2,703 ± 1,395	3,098 ± 360
	Monocytes	147–601	251 ± 149	397 ± 211
LBMPL	Leukocytes	7,408–14,440	8,750 ± 2,162	9,886 ± 2,060
	Neutrophils	3,839–9,379	5,585 ± 1,814	5,696 ± 1,523
	Eosinophils	150–709.4	295 ± 281	189 ± 86*
	Lymphocytes	2,299–5,119	2,765 ± 586	2,815 ± 565
	Monocytes	147–601	200 ± 107	364 ± 113

Absolute values (mean ± SD) of the white blood cells (WBC) count of dogs naturally infected by *Leishmania infantum* before and after receiving immunotherapy with LBMPL vaccine and/or MPL adjuvant alone. Significant differences (*P* < 0.05) are represented by the letter “^a^” related to time T0. The “*” denotes a significant difference (*P* < 0.05) between groups LBMPL and MPL at T90.

**Table 2:** **RBC of dogs naturally infected by *L. infantum* before and after immunotherapy**.

	Erythrogram	Normal range	Timeline after immunotherapy	
			T0	T90
MPL	Erythrocytes (10^6^/mm^3^)	5.42–8.38	6.0 ± 1.6	5.3 ± 0.8
	Hemoglobin (g/dL)	13.2–21.2	12.8 ± 3.7	12.1 ± 2.2
	Hematocrit (%)	33.7–57.3	35.2 ± 9.1	31.8 ± 4.5
	Platelets (10^3^/mm^3^)	171–384	135 ± 13	113 ± 46
LBMPL	Erythrocytes (10^6^/mm^3^)	5.42–8.38	4.5 ± 0.6	6.1 ± 1.1^a^
	Hemoglobin (g/dL)	13.2–21.2	11.0 ± 2.2	14.6 ± 2.4
	Hematocrit (%)	33.7–57.3	32.1 ± 6.2	37.6 ± 6
	Platelets (10^3^/mm^3^)	171–384	148 ± 45	214 ± 45*

Absolute values (mean ± SD) of the red blood cells (RBC) count of dogs naturally infected by *Leishmania infantum* before and after receiving immunotherapy with LBMPL vaccine and/or MPL adjuvant alone. Significant differences (*P* < 0.05) are represented by the letter “^a^” related to time T0. The “*” denotes a significant difference (*P* < 0.05) between groups LBMPL and MPL at T90. Values in “red” represent values below the normal range.

**Figure 1:**
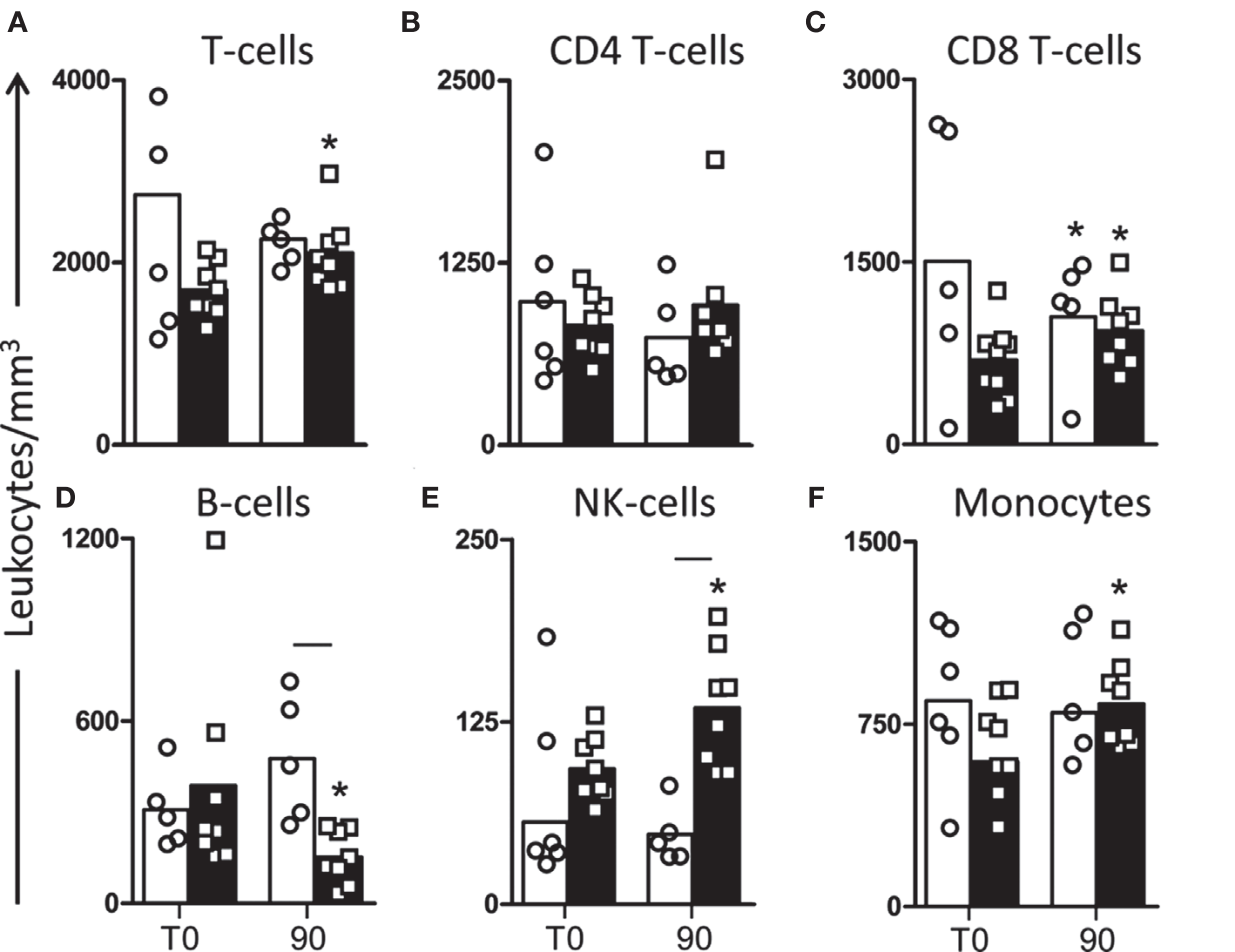
**Profile of peripheral blood leukocytes in dogs naturally infected by *Leishmania infantum* after immunotherapy with LBMPL vaccine or with MPL adjuvant alone**. The immunophenotyping of peripheral blood was used to characterize the systemic cellular profile of circulating leukocytes in dogs submitted to immunotherapy with LBMPL vaccine (black rectangle) or MPL adjuvant alone (white rectangle), before (T0) and after 90 days (T90) of treatment. Data are reported as number of leukocytes in cubic meters of blood. Results are expressed as mean values ± SD of T cells (CD3^+^) **(A)** and subpopulations CD4^+^ **(B)** and CD8^+^ **(C)**, B cells (CD21^+^) **(D)**, NK cells (CD5^−^CD16^+^) **(E)**, and monocytes (CD14^+^) **(F)**. Significant differences (*P* < 0.05) are shown by connecting line—representing differences between the LBMPL and MPL groups—and by the “*”—representing differences between T0 and T90.

In order to evaluate the immunophenotypic profile of the treated dogs in peripheral blood, we evaluated the frequency of T lymphocytes (CD3^+^) and their major subpopulations (CD4^+^ and CD8^+^) (Figures 1A–C). Our results revealed an increase (*P* < 0.05) in the number of circulating CD3^+^ T lymphocytes in dogs treated with LBMPL at T90 compared with T0 (Figure 1A). Moreover, we observed an increase (*P* < 0.05) in the number of circulating CD8^+^ T lymphocytes in dogs of LBMPL at T90 when compared with T0 and reduced number (*P* < 0.05) of these cells in MPL dogs at T90 when compared with T0 (Figure 1C). In addition, we observed a relevant increase (*P* < 0.05) in NK cells after treatment (T90) in LBMPL dogs compared with MPL group and with T0 (Figure 1E). Similarly, LBMPL animals showed a significant reduction (*P* < 0.05) in CD21^+^ B lymphocytes at T90 compared with MPL group and compared with T0 (Figure 1D). When we evaluated the levels of potential antigen-presenting cells, only dogs treated with the LBMPL vaccine demonstrated higher number (*P* < 0.05) of circulating CD14^+^ monocytes at T90 compared with T0 (Figure 1F).

### LBMPL Immunotherapy Triggers an Intense *In Vitro* Cell Proliferation and Secretion of Cytokines in the Presence of Antigenic Stimuli

**Figure 2:**
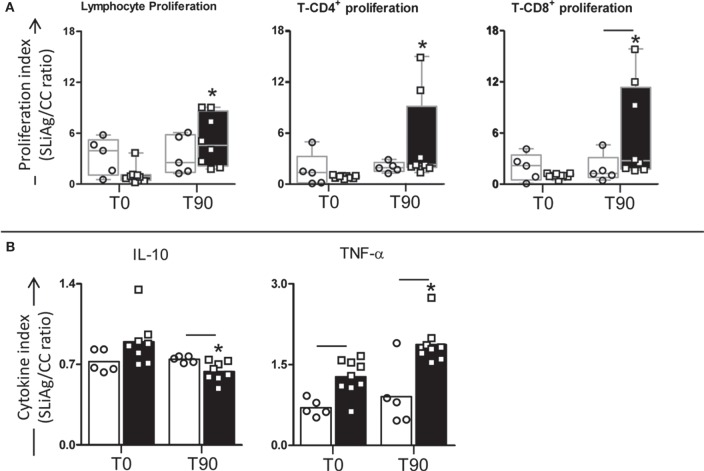
**Profile of lymphocyte proliferation and cytokine production in peripheral blood mononuclear cells (PBMCs) of dogs naturally infected by *Leishmania infantum* after immunotherapy with LBMPL vaccine or with MPL adjuvant alone**. The lymphocyte proliferation response and cytokine production were evaluated in PBMCs after *in vitro* stimulation with soluble *Leishmania infantum* antigen (SLiAg) in dogs submitted to immunotherapy with LBMPL vaccine (black rectangle) or MPL adjuvant alone (white rectangle); before (T0) and after 90 days (T90) of treatment. Lymphocyte proliferation **(A)** and cytokine production **(B)** indexes were calculated as the proportion of lymphocyte, T-CD4^+^ and T-CD8^+^ proliferation **(A)** and IL-10 and TNF-α cytokine production **(B)** observed in SLiAg-stimulated cultures divided by the control culture (SLiAg/CC ratio). The results of lymphocyte proliferation are expressed by median, and the results of cytokine production are expressed by mean. Significant differences (*P* < 0.05) are shown by connecting lines–representing differences between the LBMPL and MPL groups—and by the “*”—representing differences between T0 and T90.

To explore the effects of LBMPL immunotherapy on *in vitro* cell proliferation, we measured lymphoproliferative activation of PBMCs after immunotherapy (Figure 2A). Comparative analysis of the different treatment groups showed a significant increase (*P* < 0.05) in the lymphocyte proliferation in LBMPL dogs at T90 compared with T0. In addition, SLiAg-T-CD4^+^ proliferation was observed only in the LBMPL group with a higher (*P* < 0.05) lymphoproliferative ratio at T90 compared with before treatment (T0) (Figure 2A). Moreover, the most significant lymphoproliferative activation was observed in SLiAg-T-CD8^+^ lymphocytes in LBMPL animals (*P* < 0.05) when compared with MPL group at T90 and compared with T0 (LBMPL) (Figure 2A).

In addition to the cell proliferation profile, we evaluated the cytokines produced by the PBMCs after SLiAg stimuli (Figure 2B). Interestingly, TNF-α showed higher levels in the LBMPL dogs before (T0) and after treatment (T90) when compared with the MPL group (*P* < 0.05). Besides that, after treatment (T90), LBMPL animals showed higher levels (*P* < 0.05) of TNF-α when compared with the T0 (Figure 2B). On the other hand, the LBMPL dogs presented lower levels of IL-10 than MPL group (*P* < 0.05) at T90 (Figure 2B). Furthermore, expressive reduction in the levels of IL-10 (*P* < 0.05) was observed in LBMPL dogs at T90 when compared with T0 (Figure 2B).

### High Intracytoplasmic Synthesis of IFN-γ^+^ by Lymphocyte Subsets (CD4^+^ and CD8^+^) after *In Vitro* Antigen-Specific Stimulation Is a Marker of Immune Restoration Promoted by LBMPL Immunotherapy

**Figure 3:**
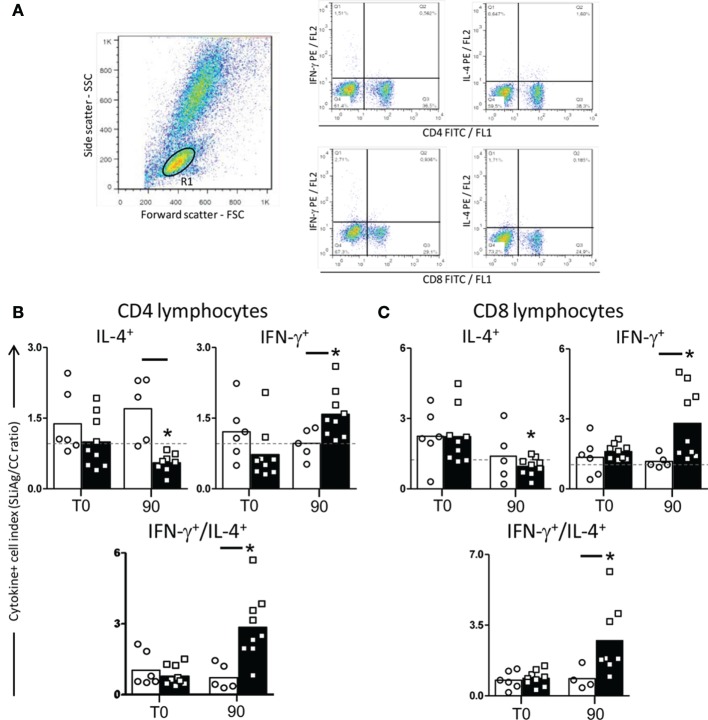
**Profile of intracytoplasmatic cytokines in peripheral blood mononuclear cells (PBMCs) of dogs naturally infected by *Leishmania infantum* after immunotherapy with LBMPL vaccine or with MPL adjuvant alone**. Pseudocolor plots illustrating the analysis of intracellular cytokines^+^ (IL-4 and IFN-γ) **(A)** in CD4^+^ cells **(B)** and CD8^+^ cells **(C)** within gated lymphocytes in PBMCs after *in vitro* stimulation with soluble *Leishmania infantum* antigen (SLiAg) in dogs submitted to immunotherapy with LBMPL vaccine (black rectangle) or MPL adjuvant alone (white rectangle); before (T0) and after 90 days (T90) of treatment. The frequency of cytokines^+^ T-cell subsets were calculated by quadrant statistics approach and first reported as percentage of gated lymphocytes prior to the calculation of the SLiAg/control indexes. The CD4^+^ and CD8^+^ cytokine indexes **(B,C)** were calculated as the proportion of cytokine^+^ cells observed in SLiAg-stimulated cultures divided by the control culture (SLAg/CC ratio). Significant differences (*P* < 0.05) are shown by connecting lines—representing differences between the LBMPL and MPL groups—and by the “*”—representing differences between T0 and T90.

In order to evaluate the cytokine profile produced by T-lymphocyte subsets, the intracytoplasmic synthesis of IFN-γ and IL-4 was assessed after antigen-specific stimulation (Figure 3A). In CD4^+^ lymphocytes, we observed reduction in IL-4 production (*P* < 0.05) in LBMPL dogs when compared with MPL group at T90. On the other hand, CD4^+^ lymphocytes showed an increase in the IFN-γ production at T90 in LBMPL dogs when compared with MPL group. Furthermore, we observed higher numbers of CD4^+^IFN-γ^+^ lymphocytes at T90 in LBMPL group when compared with the time before treatment (T0) (Figure 3B). Based on CD8^+^ lymphocytes analysis, we observed higher numbers of CD8^+^IFN-γ^+^ cells (*P* < 0.05) in LBMPL dogs when compared with MPL group at T90 and compared with T0 (LBMPL) (Figure 3C).

We also evaluated the IFN-γ/IL-4 ratio of the T-lymphocyte subsets and that was higher (*P* < 0.05) in LBMPL dogs than in the MPL group at T90 for both, CD4^+^ and CD8^+^ lymphocytes (Figures 3B,C). Similarly, there was a consistent higher IFN-γ/IL-4 ratio in LBMPL group (*P* < 0.05) after treatment (T90) when compared with T0 (Figures 3B,C).

### LBMPL Immunotherapy Leads to an Important Parasite Reduction in Bone Marrow and Skin with Block Transmission after Xenodiagnosis

**Figure 4:**
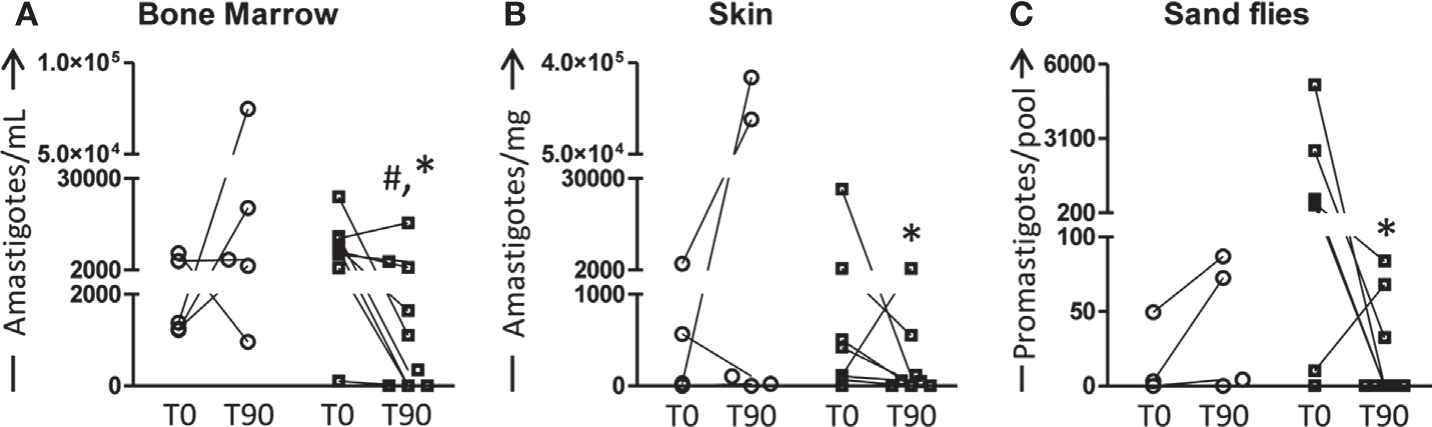
**Quantification of parasite burden in bone marrow, skin, and sand flies of dogs naturally infected by *Leishmania infantum* after immunotherapy with LBMPL vaccine or with MPL adjuvant alone**. The parasite burden was performed to evaluate the curative properties of LBMPL vaccine (black and white squares) or MPL adjuvant alone (white circle); before (T0) and after 90 days (T90) of treatment. The number of amastigotes was estimated in bone marrow **(A)**, skin **(B)**, and sand flies **(C)**. Results were plotted representing individual values of each dog per group with connecting lines of T0 and T90. Significant differences (*P* < 0.05) are shown by “^#^”—representing difference between the LBMPL and MPL groups—and by the “*”—representing differences between T0 and T90.

The main parasitological features presented by all animals are summarized in Figure 4. As shown in Figure 4A, low number (*P* < 0.05) of amastigotes was observed in the bone marrow of LBMPL group when compared with MPL dogs at T90. Moreover, this number of parasites was lower (*P* < 0.05) compared with the time before treatment (T0) in the LBMPL dogs (Figure 4A). When we evaluated the skin, only LBMPL group showed an important reduction (*P* < 0.05) in the number of amastigotes at T90 when compared with T0 (Figure 4B). Moreover, when we assessed the parasite transmission to sand flies through xenodiagnosis, the LBMPL group induced an important reduction (*P* < 0.05) in the capacity to infect sand flies after treatment (T90) when compared with before treatment (T0). Furthermore, in the LBMPL group, six of nine dogs showed parasites after xenodiagnosis at T0. On the other hand, only three dogs demonstrated parasites after xenodiagnosis at T90. These data are associated with a negative detection of parasites in 33, 44, and 67% in LBMPL dogs in bone marrow, skin, and sand flies, respectively (data not shown).

### VL Dogs Exhibit Clinical Improvement and Reduction of Splenomegaly after Immunotherapy with LBMPL Vaccine

**Figure 5:**
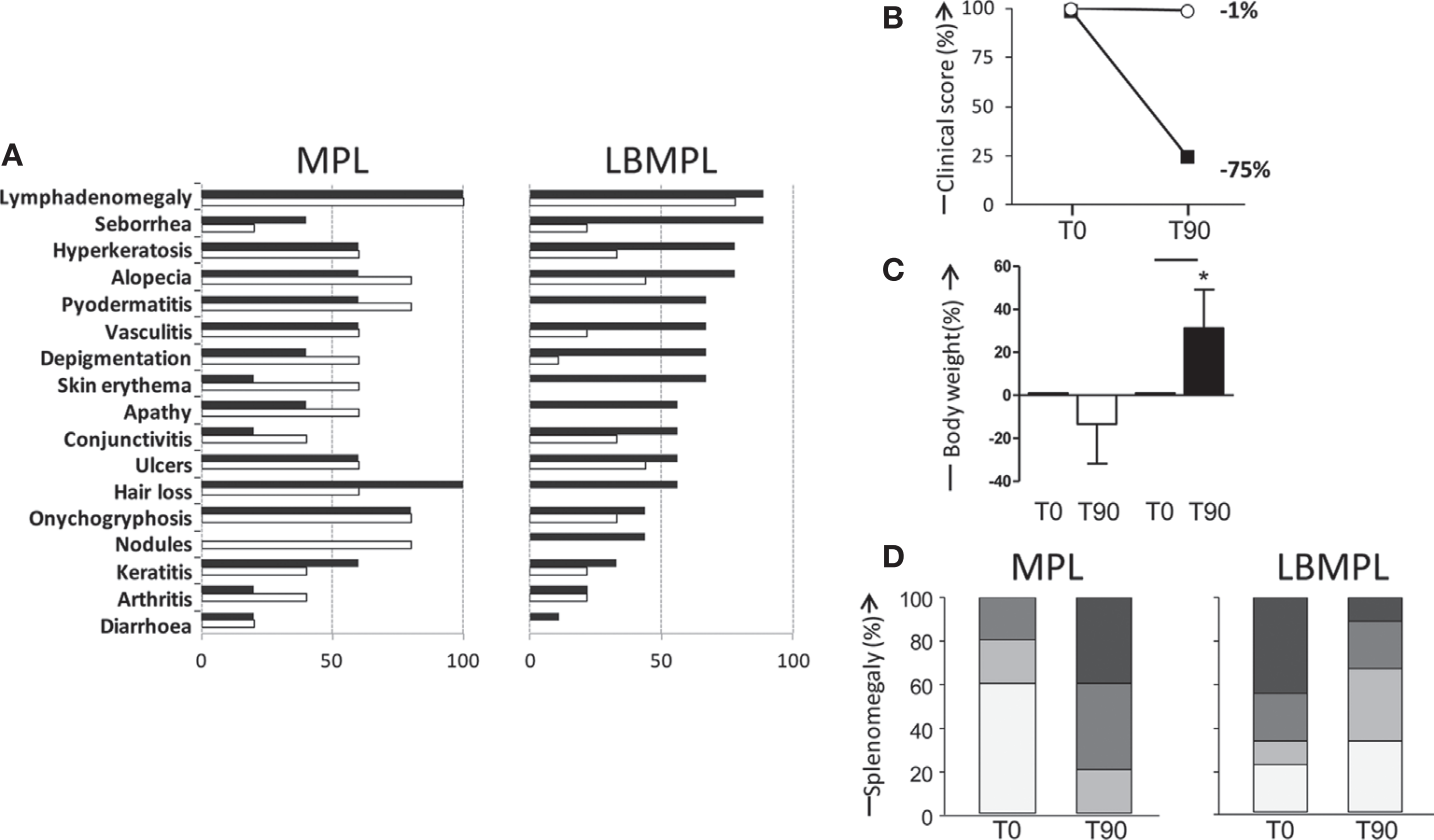
**Evaluation of clinical outcome [visceral leishmaniasis (VL) signs], clinical score, body weight, and splenomegaly of dogs naturally infected by *Leishmania infantum* after immunotherapy with LBMPL vaccine or with MPL adjuvant alone**. **(A)** Percentage of dogs with suggested VL signs submitted to immunotherapy with LBMPL vaccine (right) or MPL adjuvant alone (left), before (T0 = black rectangle) and after 90 days (T90 = white rectangle) of treatment. **(B)** Representative graph of the percentage of reduction of clinical score in dogs submitted to immunotherapy with LBMPL vaccine (black square) or MPL adjuvant alone (white circle), before (T0) and after 90 days (T90) of treatment. **(C)** Percentage of gain weight in dogs submitted to immunotherapy with LBMPL vaccine (black rectangle) or MPL adjuvant alone (white rectangle), before (T0) and after 90 days (T90) of treatment. **(D)** Percentage of animals classified according to the absence of splenomegaly (normal = white rectangle), mild splenomegaly (light gray), moderate (medium gray), and severe (dark gray); in dogs submitted to immunotherapy with LBMPL vaccine (right) or MPL adjuvant alone (left), before (T0) and after 90 days (T90) of treatment. Significant differences (*P* < 0.05) are shown by connecting lines—representing differences between the LBMPL and MPL groups—and by the “*”—representing differences between T0 and T90.

Before treatment—T0, all animals presented several clinical signs of CVL, and these signs were used as inclusion criteria. Figure 5A showed the main signs of CVL presented before (T0) and after (T90) the LBMPL and MPL treatment. In MPL dogs, we observed a disease progression with increase of the signs of CVL such as, seborrheic dermatitis, alopecia, pyodermatitis, skin erythema, depigmentation, apathy, conjunctivitis, nodules, and arthritis (Figure 5A). On the other hand, in dogs submitted to LBMPL immunotherapy, we observed reduction on signs of CVL such as, lymphadenomegaly, seborrheic dermatitis, hyperkeratosis, and alopecia. Moreover, many clinical signs of disease were not observed after LBMPL treatment, for example, pyodermatitis, skin erythema, apathy, hair loss, nodules, and diarrhea (Figure 5A). Dogs in the LBMPL-treated group presented a general improvement in their clinical score with reduction of intensity and number of CVL signs with a percentage of reduction of 75% (Figure 5B). In contrast, the dogs of the MPL group presented an aggravation of the clinical status during all treatment period with only 1% of reduction of their clinical score and one dog from the MPL group died due to cachexia (Figure 5B). Besides that, we observed a body weight gain (*P* < 0.05) in the LBMPL dogs after treatment (T90) compared with T0 (Figure 5C). On the other hand, the MPL group showed a significant reduction (*P* < 0.05) of body mass at T90 suggesting progression of disease (Figure 5C). Using ultrasound equipment, we evaluated the spleen size in MPL and LBMPL dogs at T0 and after immunotherapy (T90). As shown in Figure 5D before the LBMPL immunotherapy, 70% of the dogs exhibited moderate/severe splenomegaly. After treatment, we observed a strong reduction of this alteration with only 30% of animals presenting moderate/severe splenomegaly. In contrast, MPL dogs showed an increase in moderate/severe splenomegaly ranging from 20% before treatment to 80% after treatment.

**Figure 6:**
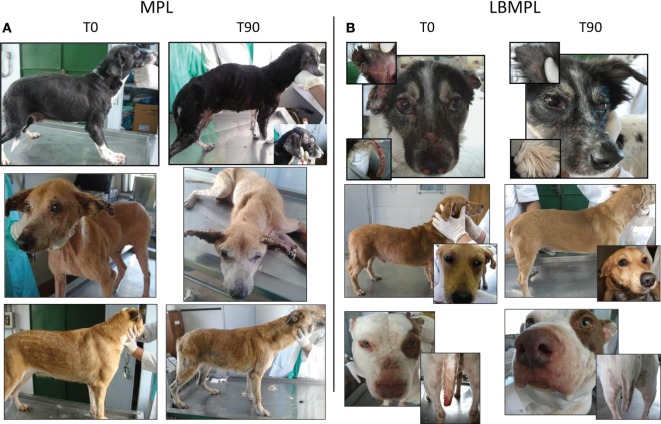
**Clinical efficacy of dogs naturally infected by *Leishmania infantum* after immunotherapy with LBMPL vaccine or with MPL adjuvant alone**. Illustrative images of dogs submitted to immunotherapy with MPL adjuvant alone **(A)** or LBMPL vaccine **(B)**, before (T0) and after 90 days (T90) of treatment.

Figure 6 shows representative examples of CVL signs of both groups, MPL and LBMPL, before (T0) and after treatment (T90). LBMPL immunotherapy promoted a substantial clinical improvement in dogs when compared with the MPL group as shown by the dogs in Figure 6B. Most of the LBMPL-treated animals showed significant clinical improvement with some dogs demonstrating complete remission of clinical signs suggestive of VL (Figure 6B). On the other hand, animals treated with MPL alone got worse their VL symptoms during and at the end of the study (Figure 6A) At the end of study, we observed that 78% (7/9) of LBMPL-treated dogs showed significant clinical improvement in relation to the signs and symptoms suggestive of VL. On the other hand, 100% (5/5) of the dogs treated with MPL showed no clinical remission of signs progressing gradually toward the disease.

## Discussion

A current problem that has worsened the situation of VL in the world is associated with the large number of non-responders patients to conventional chemotherapy with antimony, suggesting the emergence of resistant strains of the parasite (41). For this reason, there is a regional variation in the response to treatment in VL patients, and recommendations for the use of conventional drugs may vary in different regions of the world (42). In this sense, the use of therapeutic vaccines and other immunobiological agents has attracted attention in the treatment for leishmaniasis. This strategy, known as immunotherapy, uses biological substances and/or immunobiological agents that act to modulate or modify the immune response in order to achieve a prophylactic and/or therapeutic objective (43). The immunotherapeutic agents may exert their effect by direct or indirect action increasing the natural defenses and restoring the disabled effectors function.

In the present work, we conducted a detailed analysis of an immunotherapy protocol using the LBMPL vaccine as potential treatment for VL, using symptomatic dogs naturally infected by *L. infantum* as experimental model. We evaluated the main hematological changes and blood leukocytes profile in dogs submitted to immunotherapy with LBMPL vaccine as well as the group treated with MPL adjuvant only. As observed, important normalization in RBC with restoration of erythrocytes, hemoglobin, hematocrit, and platelets was achieved after immunotherapy. Moreover, after blood evaluation, the immune profile observed in treated dogs showed an increase in CD3^+^ T cells represented mainly by CD8^+^ T-cell subpopulation, increase in NK cells and CD14^+^ monocytes and decrease in B-cells. Patients with active and severe VL exhibit a blood marker anemia with decreased values of hemoglobin and hematocrit and low counts of platelets (44). For both, human and dogs, the decline of these parameters indicates an adverse prognosis. As we observed, the immunotherapy with LBMPL vaccine promoted normalization in these biomarkers. Furthermore, restoration of the circulating leukocytes, mainly the specific T cells and monocytes, reinforces a beneficial nature of the immunotherapeutic treatment performed. Many studies using vaccines and other immunomodulators show that the restoration of circulating T lymphocytes with others cells is essential to clinical improvement and control of parasitism (24, 45, 46).

The activation and enhancement of an effective cell immune response are the challenges associated with severe VL. In our study, we evaluated whether the immunotherapy with LBMPL would activate PBMCs after *in vitro* antigenic stimulation with SLiAg. Our results showed that higher cell reactivity after stimulation with SLiAg was recorded in the LBMPL-treated dogs accompanied by increased lymphoproliferation. Also, this increase was marked by both specific CD4^+^ and CD8^+^ *L. infantum*-T lymphocytes. The positive lymphoproliferative immune response against the *Leishmania* antigen is a classic marker of resistance in both canine and human disease (15, 47–49). It has been reported that naturally infected dogs presenting no clinical signs showed 98% of positive correlation between intradermal response (IDR) and *in vitro* lymphoproliferative activation (50). In some patients with diffuse cutaneous leishmaniasis was observed a positive IDR with *Leishmania* antigens after immunotherapy with pasteurized *L. braziliensis* vaccine plus BCG and, even 10 months after treatment, all those patients presented remission of symptoms indicating clinical cure against the disease (51). Associated with *in vitro* lymphoproliferative response, we observed a strong TNF-α production with low levels of IL-10, an immunomodulatory cytokine. These results confirm the effect promoted by LBMPL immunotherapy activating a pro-inflammatory cell immune response consistent with the development of a protective immunity against the disease.

Considering the combined analysis of T-cell subpopulations and cytokine production, both CD4^+^ and CD8^+^ T cells are relevant sources of IFN-γ after treatment with LBMPL vaccine. On the other hand, these cells did not produce or secrete IL-4. As shown classically, the increased production of IFN-γ is critical for parasitism control through microbicidal activity of macrophages (52). Regarding IL-4, it has been suggested that this cytokine acts by inhibiting transduction of signals for inducible nitric oxide synthase reducing the NO production by macrophages (53). In a VL murine model, the immunotherapy using liposomal resiquimod promoted an increase in IFN-γ levels and as consequence, the tissue parasitism control (54). Dealing with the natural infection, it is observed in asymptomatic dogs a resistant immune profile characterized by high levels of both IFN-γ and TNF-α and low levels of IL-10 (55). As described elsewhere, after successful VL treatment patients gradually regain their cells capacity to produce IFN-γ, but not IL-10 after specific *Leishmania* stimuli (56). In this context, our results suggest that immunotherapy with LBMPL vaccine was able to promote in the dogs a consistent immune activation with production of inflammatory cytokines (IFN-γ and TNF-α) and low production of immunomodulatory cytokines characterizing a control profile of the disease.

Following treatment with LBMPL vaccine, dogs demonstrated a pronounced improvement of clinical signs and in the clinic pathological abnormalities. Moreover, the improvement observed in LBMPL dogs was gradual and included complete regression or marked reduction in the number and degree of skin alterations, weight gain, reduction and/or absence of lymphadenopathy, restoration in the color of the mucous membranes, and regression in the eye lesions. This reduction of clinical signs in dogs represented 75% decrease in the clinical score in dogs of LBPML group and some animals presenting no CVL alterations with total clinical cure at the final of the experiment. The use of an ultrasound device helped with the splenomegaly evaluation and we observed a normalization of the spleen size in more than 60% of LBMPL-treated dogs compared with MPL control animals (only 20%). Furthermore, dogs submitted to immunotherapy with LBMPL vaccine showed no signs of pain at the site of injection and no side effects. Miret et al. (22) using a vaccine combined with MPL-SE (Leish-110f^®^ + MPL-SE^®^) in dogs observed improvement in clinical response after immunotherapy. On the other hand, when MPL-SE^®^ was used, dogs got worse their symptoms during and at the end of the study (22). Using a protein aggregate magnesium–ammonium phospholinoleate–palmitoleate anhydride immunomodulator (P-MAPA), Santiago et al. (24) observed an intense improvement in clinical signs of dogs submitted to this immunotherapy. On the other hand, as observed in our study, clinical signs worsened in control group with dogs presenting intense loss of weight, anorexia, and cachexia (24). Regarding human VL, pioneering studies conducted by Badaro et al. (15) evaluating immunochemotherapy protocol with meglumine antimoniate and IFN-γ in patients with therapeutic failure and/or severe VL showed 90% of clinical cure after treatment. These authors suggest that the high rates of clinical cure achieved were largely due to immunotherapy protocol used (15).

Often, clinical improvement observed in treated animals and patients with VL is accompanied by parasitism reduction. In our study, we evaluated the parasite burden in bone marrow, skin, and sand flies after xenodiagnosis using the qPCR. As observed, dogs immunotreated with LBMPL vaccine presented an intense parasite reduction in both bone marrow and skin before and after treatment. da Silva et al. (36) evaluating a combined therapy using liposomal meglumine antimoniate and allopurinol in VL dogs, observed a significant reduction in the parasite load at spleen and liver after treatment. Similarly, Manna et al. (57) observed reduction in parasite load in the spleen and lymph nodes following administration of miltefosine in combination with allopurinol in CVL animals. Similar results were obtained by Miret et al. (22) using conventional parasitological tests (smear and culture) to evaluate immunotreated dogs. All dogs treated with vaccine had negative parasitological tests. On the other hand, the control adjuvant group (MPL-SE^®^) presented positive parasitological tests in all dogs in bone marrow and skin (22). Evaluating the skin, the most important tissue and source of parasite transmission to the vectors, we observed a significant decrease in parasite load in LBMPL-treated dogs with 127 times lower parasite burden compared with the time before treatment. As discussed by some authors, one of the most important objectives of the CVL treatment is the blockade of transmission to sand flies by reducing the parasite load in the skin concomitantly with decrease of the disease and remission of symptoms (58). Therefore, we evaluated the ability of the LBMPL-treated dogs to infect sand flies by xenodiagnosis. It is possible to observe in our study that LBMPL vaccine therapy was capable to promote a significant reduction in the parasite infection to sand flies. Thus, both the skin parasite load and xenodiagnosis results showed that the immunotherapy with LBMPL vaccine was effective, controlling the parasite burden in the skin associated with reduction transmission to *L. longipalpis*.

As observed in some studies, depending on the clinical condition of human patients and dogs, the use of chemotherapy is inefficient (59, 60), and the association of immunomodulators and or vaccines would promote an effective cellular immune response controlling the parasite proliferation. As noted in our study, the immunotherapy using LBMPL vaccine in symptomatic VL dogs promoted the normalization of hematological and biochemical parameters, the restoration, activation, and polarization of the immune response with a protective profile, clinical improvement, and intense reduction in the parasite burden with potential to block transmission to sand flies. The encouraging results presented here reinforce the relevance of previous studies in CVL that can indicate immunotherapy as significant approach to be used as potential treatment strategy. Our work can be seen as a subsidy for future studies to evaluate the use of LBMPL combined with other leishmanicidal drugs (immunochemotherapy), aiming the reduction of (i) administration period, (ii) dose of the drug required, and (iii) side effects. Based on that, it will be possible to analyze if this combination can promote more effectively clinical and parasitological cure in VL.

## Author Contributions

BR, RG, and AR designed the study. BR, LR, FM, SS, and SF assisted in conducting and performed the experiments. BR, RA-S, JC, JV, and AR analyzed and interpreted the data. NG and RC-O supplied additional reagents for this study. BR and AR wrote the manuscript. All authors have read, reviewed, and edited the manuscript and agreed for submission to this journal.

## Conflict of Interest Statement

The authors have declared that there are no competing interests. This work is related to the pendent Patent BR1020130237680, INPI, Brazil.
