# Supplementary figures and images for: A Vaccine Therapy for Canine Visceral Leishmaniasis Promoted Significant Improvement of Clinical and Immune Status with Reduction in Parasite Burden
Source: Front Immunol. 2017 Mar 7;8:217. doi: 10.3389/fimmu.2017.00217 (PMC5338076; doi:10.3389/fimmu.2017.00217)

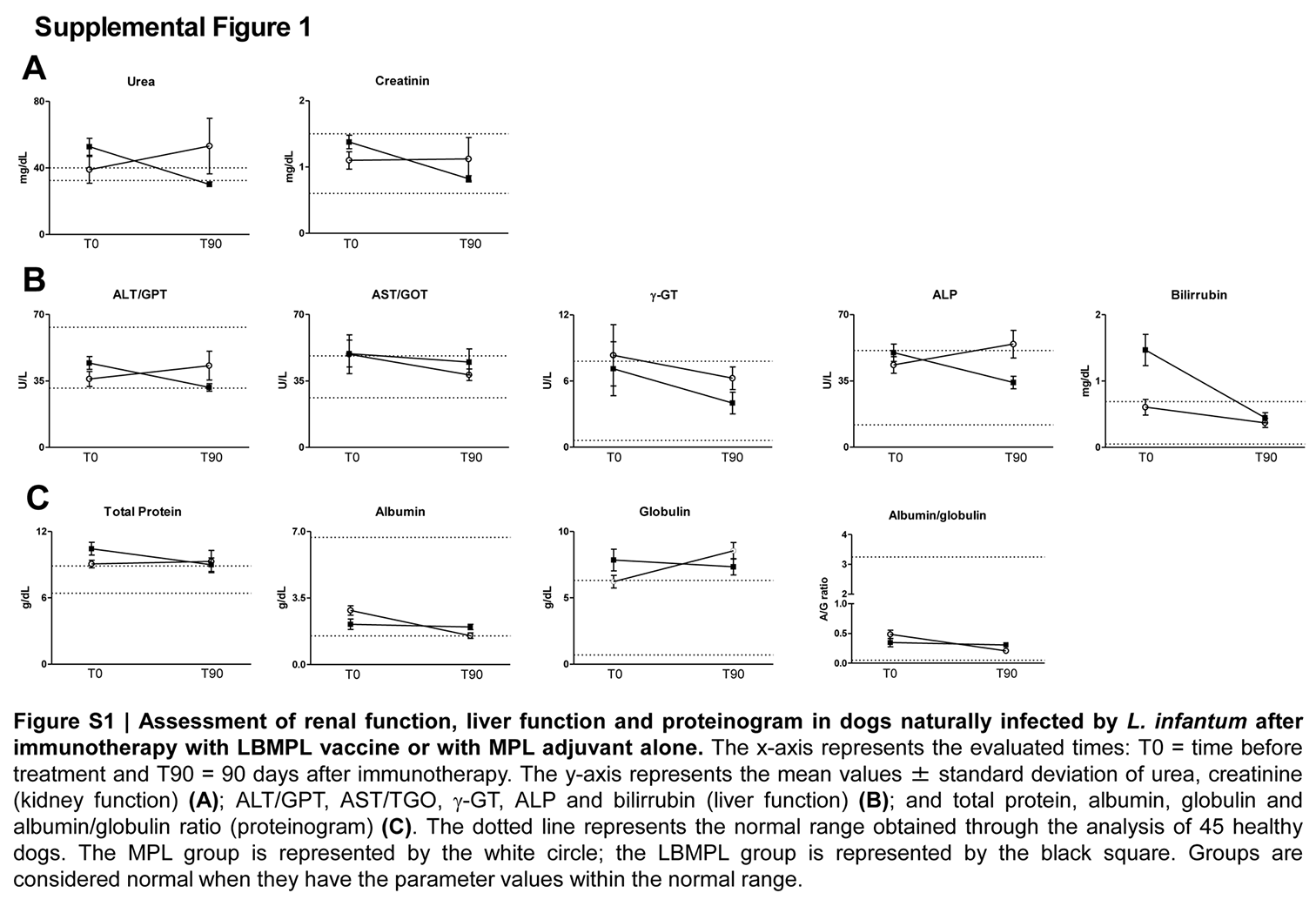

Supplement: Supplementary file 1 [file image_1.tif]
